# Supplementary material for: Cost components of school-based oral health-promoting programs: A systematic review protocol
Source: PLoS One. 2023 Sep 28;18(9):e0287244. doi: 10.1371/journal.pone.0287244 (PMC10538792; doi:10.1371/journal.pone.0287244)
Supplement: S1 File — (DOCX) [file pone.0287244.s002.docx]

**S2 – Search strategies**

| **SOURCE OF INFORMATION** | **SEARCH STRATEGY** |
| --- | --- |
| **MEDLINE**  **(PUBMED)** | **(("students"[MeSH Terms] OR "child"[MeSH Terms] OR "adolescent"[MeSH Terms] OR "student"[Title/Abstract] OR "school enrolment"[Title/Abstract] OR "school enrollments"[Title/Abstract] OR "school child"[Title/Abstract] OR "school children"[Title/Abstract] OR "schooler"[Title/Abstract] OR "schoolers"[Title/Abstract] OR "teen"[Title/Abstract] OR "teens"[Title/Abstract] OR "teenager"[Title/Abstract] OR "teenagers"[Title/Abstract]) AND ("Oral Health"[MeSH Terms] OR "Dental Care"[MeSH Terms] OR "Dental Care for Children"[MeSH Terms] OR "Dental Health Services"[MeSH Terms] OR "School Dentistry"[MeSH Terms] OR "School Health Services"[MeSH Terms] OR "Toothbrushing"[MeSH Terms] OR "Dental Atraumatic Restorative Treatment"[MeSH Terms] OR "health education, dental"[MeSH Terms] OR "Preventive Dentistry"[MeSH Terms] OR "Healthy People Programs"[MeSH Terms] OR "Health Promotion"[MeSH Terms] OR ((((((((("dentistry for children"[Title/Abstract] OR "dental health service"[Title/Abstract] OR "school health service"[Title/Abstract] OR "school based services"[Title/Abstract] OR "school based services"[Title/Abstract] OR "school based service"[Title/Abstract] OR "school based health services"[Title/Abstract] OR "school based health services"[Title/Abstract] OR "school based health service"[Title/Abstract] OR "school health promotion"[Title/Abstract]) AND ("health education, dental"[MeSH Terms] OR ("Health"[All Fields] AND "education"[All Fields] AND "Dental"[All Fields]) OR "dental health education"[All Fields] OR ("Dental"[All Fields] AND "Health"[All Fields] AND "education"[All Fields]))) AND "promotion of health"[Title/Abstract]) AND ("Health Promotion"[MeSH Terms] OR ("Health"[All Fields] AND "Promotion"[All Fields]) OR "Health Promotion"[All Fields] OR ("Promotional"[All Fields] AND "items"[All Fields]) OR "promotional items"[All Fields])) AND "promotional item"[Title/Abstract]) AND ("wellness programmes"[All Fields] OR "Health Promotion"[MeSH Terms] OR ("Health"[All Fields] AND "Promotion"[All Fields]) OR "Health Promotion"[All Fields] OR ("Wellness"[All Fields] AND "programs"[All Fields]) OR "wellness programs"[All Fields])) AND "wellness program"[Title/Abstract]) AND ("Health Promotion"[MeSH Terms] OR ("Health"[All Fields] AND "Promotion"[All Fields]) OR "Health Promotion"[All Fields] OR ("Health"[All Fields] AND "campaigns"[All Fields]) OR "health campaigns"[All Fields])) AND "health campaign"[Title/Abstract]))) AND ("Costs and Cost Analysis"[MeSH Terms] OR (("Costs"[Title/Abstract] AND "cost analyses"[Title/Abstract]) OR "Cost"[Title/Abstract] OR "Costs"[Title/Abstract] OR "cost analysis"[Title/Abstract] OR "cost analyses"[Title/Abstract] OR "cost measures"[Title/Abstract] OR "cost measure"[Title/Abstract] OR "Pricing"[Title/Abstract] OR "Affordability"[Title/Abstract] OR "cost comparison"[Title/Abstract] OR "cost comparisons"[Title/Abstract] OR "economic evaluation"[Title/Abstract] OR "economic evaluations"[Title/Abstract] OR "cost assessment"[Title/Abstract] OR "cost assessments"[Title/Abstract]))** |
| **EMBASE** | **(('student'/exp OR 'middle school student'/exp OR 'elementary student'/exp OR 'school child'/exp OR students:ti,ab,kw OR 'school enrollment':ti,ab,kw) AND ('dental procedure'/exp OR 'school dentistry'/exp OR 'school health service'/exp OR 'tooth brushing'/exp OR 'atraumatic restorative treatment'/exp OR 'atraumatic technique'/exp OR 'health education'/exp OR 'preventive dentistry'/exp OR 'health promotion'/exp OR 'school health education'/exp OR 'wellness center'/exp) OR 'oral health care':ti,ab,kw OR 'dental care for children':ti,ab,kw OR 'dental health':ti,ab,kw OR 'healthy people programs':ti,ab,kw OR 'dentistry for children':ti,ab,kw OR 'oral health':ti,ab,kw OR 'dental care':ti,ab,kw OR 'promotional items':ti,ab,kw OR 'promotion of health':ti,ab,kw) AND ('cost'/exp OR 'health economics'/exp OR 'economic evaluation'/exp OR 'cost benefit analysis'/exp) AND [embase]/lim** |
| **WEB OF SCIENCE (CORE COLLECTION)** | **(((TS=((("students" OR "child" OR "adolescent" OR "student" OR "school enrolment" OR "school enrollments" OR "school child" OR "school children" OR "schooler" OR "schoolers" OR "teen" OR "teens" OR "teenager" OR "teenagers")) )) AND TS=((("Oral Health" OR "Dental Care" OR "Dental Care for Children" OR "Dental Health Services" OR "School Dentistry" OR "School Health Services" OR "Toothbrushing" OR "Dental Atraumatic Restorative Treatment" OR "dental health education" OR "Preventive Dentistry" OR "Healthy People Programs" OR "Health Promotion" OR "dentistry for children" OR "dental health service" OR "school health service" OR "school based services" OR "school based services" OR "school based service" OR "school based health services" OR "school based health services" OR "school based health service" OR "school health promotion" OR "dental health education" OR "promotion of health" OR "Health Promotion" OR "promotional items" OR "wellness programmes" OR "Health Promotion" OR "wellness programs" OR "wellness program" OR "health campaigns" ))))) AND TS=((("Costs and Cost Analysis" OR "Costs" OR "Cost" OR "Costs" OR "cost analysis" OR "cost analyses" OR "cost measures" OR "cost measure" OR "Pricing" OR "Affordability" OR "cost comparison" OR "cost comparisons" OR "economic evaluation" OR "economic evaluations" OR "cost assessment" OR "cost assessments")))** |
| **SCOPUS** | **( TITLE-ABS-KEY ( students OR child OR & AND adolescent OR student OR "school enrollment" OR "school enrollments" OR "school child" OR "school**  **children" OR schooler OR schoolers OR teen OR teens OR teenager OR teenagers ) ) AND ( TITLE-ABS-KEY ( "oral health" OR "dental care" OR "dental care for children" OR "dental health services" OR "school dentistry" OR "school health services" OR "toothbrushing" OR "dental atraumatic restorative treatment" OR "dental health education" OR "preventive dentistry" OR "healthy people programs" OR "health promotion" OR "dentistry for children" OR "dental health service" OR "school health service" OR "school-based services" OR "school based services" OR "school-based service" OR "school-based health services" OR "school based health services" OR "school-based health service" OR "school health promotion" OR "dental health education" OR "promotion of health" OR "promotional items" OR "promotional item" OR "wellness programs" OR "wellness program" OR "health campaigns" OR "health campaign" ) ) AND ( TITLE-ABS-KEY ( "costs and cost analysis" OR "costs and cost analyses" OR cost OR "cost analysis" OR "cost analyses" OR "cost measures" OR "cost measure" OR "pricing" OR "affordability" OR "cost comparison" OR "cost comparisons" OR "economic evaluation" OR "economic evaluations" OR "cost assessment" OR "cost assessments" ) )** |
| **COCHRANE LIBRARY** | **((students OR child OR adolescent OR student OR "school enrollment" OR "school enrollments" OR "school child" OR "school children"  OR schooler OR schoolers OR teen OR teens OR teenager OR teenagers)):ti,ab,kw AND (("oral health" OR "dental care" OR "dental care for children" OR "dental health services" OR "school dentistry" OR "school health services" OR "toothbrushing" OR "dental atraumatic restorative treatment" OR "dental health education" OR "preventive dentistry" OR "healthy people programs" OR "health promotion" OR "dentistry for children" OR "dental health service" OR "school health service" OR "school-based services" OR "school based services" OR "school-based service" OR "school-based health services" OR "school based health services" OR "school-based health service" OR "school health promotion" OR "dental health education" OR "promotion of health" OR "promotional items" OR "promotional item" OR "wellness programs" OR "wellness program" OR "health campaigns" OR "health campaign" )):ti,ab,kw AND (( "costs and cost analysis" OR "costs and cost analyses" OR cost OR "cost analysis" OR "cost analyses" OR "cost measures" OR "cost measure" OR "pricing" OR "affordability" OR "cost comparison" OR "cost comparisons" OR "economic evaluation" OR "economic evaluations" OR "cost assessment" OR "cost assessments" )):ti,ab,kw** |
| **HVL** | **(students OR estudiantes OR estudantes OR child OR niño OR criança OR adolescent OR adolescente OR "School Enrollment" OR "School Enrollments" OR student OR estudiante OR estudante OR children OR niños OR crianças OR adolescents OR adolescentes OR alumno OR alumnos OR aluno OR alunos OR "school child" OR "school children" OR "Schooler" OR "Schoolers" OR teen OR tens OR teenager OR teenagers) AND (("Oral Health" OR "Dental Care" OR "Dental Care for Children" OR "Dental Health Services" OR "School Dentistry" OR "School Health Services" OR "Toothbrushing" OR "Dental Atraumatic Restorative Treatment" OR "Dental Health Education" OR "Preventive Dentistry" OR "Healthy People Programs" OR "Health Promotion" ) OR ("Salud Bucal" OR "Saúde Bucal" OR "Atención Odontológica" OR "Assistência Odontológica" OR "Atención Dental para Niños" OR "Assistência Odontológica para Crianças" OR "Servicios de Salud Dental" OR "Serviços de Saúde Bucal" OR "Servicios de Salud Escolar" OR "Serviços de Saúde Escolar" OR "Cepillado Dental" OR "Escovação Dentária" OR "Tratamiento Restaurativo Atraumático Dental" OR "Tratamento Dentário Restaurador sem Trauma" OR "Educación en Salud Dental" OR "Educação em Saúde Bucal" OR "Odontología Preventiva" OR "Odontologia Preventiva" OR "Programas Gente Sana" OR "Programas Gente Saudável" OR "Promoción de la Salud" OR "Promoção da Saúde") OR ("Dentistry for Children" OR "Dental Health Service" OR "School Health Service" OR "School-Based Services" OR "School Based Services" OR "School-Based Service" OR "School-Based Health Services" OR "School Based Health Services" OR "School-Based Health Service" OR "School Health Promotion" OR "Promotion of Health" OR "Promotional Items" OR "Promotional Item" OR "Wellness Programs" OR "Wellness Program" OR "Health Campaigns" OR "Health Campaign" OR "Servicios de Odontología para Niños" OR "Odontologia para Crianças" OR "Servicio de Salud Dental" OR "Serviço de Saúde Bucal" OR "Servicio de Salud Escolar" OR "Serviço de Saúde Escolar" OR "Promoción de la Salud Escolar" OR "Promoción de la Salud Estudantil" OR "Promoción de la Salud en el Ambiente Escolar" OR "Promoción de la Salud en el Medio Escolar" OR "Promoción de la Salud en la Escuela" OR "Promoción de la Salud en las Escuelas" OR "Servicio de Salud Basado en la Escuela" OR "Servicios de Salud Basados en la Escuela" OR "Promoção da Saúde Escolar" OR "Promoção da Saúde dos Alunos" OR "Promoção da Saúde dos Estudantes" OR "Promoção da Saúde em Ambiente Escolar" OR "Promoção da Saúde em Meio Escolar" OR "Promoção da Saúde na Escola" OR "Promoção da Saúde no Ambiente Escolar" OR "Promoção da Saúde no Meio Escolar" OR "Serviço de Saúde Baseados na Escola" OR "Serviços de Saúde Baseados na Escola" OR "Campanas de Salud" OR "Item Promocional" OR "Items Promocionales" OR "Programas de Bienestar" OR "Promoción del Bienestar" OR "Campanhas de Saúde" OR "Itens Promocionais" OR "Programas de Bem-Estar" OR "Promoção do Bem Estar" OR "Promoção em Saúde" )) AND ("Costs and Cost Analysis" OR “costos y análisis de costo” OR “custos e análise de custo” OR costs OR "Cost Analyses" OR cost OR "Cost Analysis" OR "Cost Measures" OR "Cost Measure" OR "Pricing" OR "Affordability" OR "Cost Comparison" OR "Cost Comparisons" OR "Economic Evaluation" OR "Economic Evaluations" OR "Cost Assessment" OR "Cost Assessments" OR asequibilidad OR asequibilidades OR costos OR costo OR "Evaluación Económica" OR "Evaluaciónes Económicas" OR "Análise de Custo em Saúde" OR "Análise de Custos" OR "Comparação de Custos" OR custo OR custos OR "Custos e Análises de Custo" OR "Medidas de Custo" OR precificação OR "Capacidade de Pagar pelos Serviços de Saúde") AND ( db:("LILACS" OR "BDENF" OR "BBO" OR "IBECS" OR "BINACIS" OR "INDEXPSI" OR "WHOLIS" OR "BRISA" OR "SES-SP" OR "MINSAPERU" OR "CUMED" OR "colecionaSUS" OR "LIPECS" OR "RHS" OR "BDNPAR"))** |
| **CRD (HTA AND NHS EED)** | **(student* OR child* OR adolescent* OR school child* OR schooler*) AND (oral health OR dental care OR Atraumatic Restorative Treatment OR toothbrusing OR dental care for child* OR school dentistry OR school health service* OR health promotion OR preventive dentistry) AND (costs and cost analysis OR cost*OR economic evaluation OR dental economics OR economics) IN NHSEED, HTA** |
