# Supplementary material for: Cost components of school-based oral health-promoting programs: A systematic review protocol
Source: PLoS One. 2023 Sep 28;18(9):e0287244. doi: 10.1371/journal.pone.0287244 (PMC10538792; doi:10.1371/journal.pone.0287244)
Supplement: S2 File — (DOCX) [file pone.0287244.s003.docx]

**S3 - Quality Assessment Tool**

| **Reviwer:** | **Date:** | | | |
| --- | --- | --- | --- | --- |
| **Author/year:** | **Record number:** | | | |
| **Items Assessed** | **Fully meet the item** | **Partially meet the item** | **Did**  **not meet the item** | **Not applicable** |
| **Was the objective of the study clearly stated and properly answered?** |  |  |  |  |
| **Was the target population of the study clearly described (e.g., gender, age group, regional distribution, or socioeconomic status)?** |  |  |  |  |
| **Was the study perspective stated?** |  |  |  |  |
| **Was there a description of the method for cost estimation?** |  |  |  |  |
| **Were the cost components included in the analysis in line with the perspective adopted in the study?** |  |  |  |  |
| **Were the cost components clearly described and presented in a disaggregated way to allow for transparency and reproducibility?** |  |  |  |  |
| **Was there information on the currency and the period in which the costs were collected clearly stated?** |  |  |  |  |
| **If the costs were collected over different periods, was there an adjustment for inflation? (Not applicable for single-year cross-sectional studies)** |  |  |  |  |
| **Were the cost components results presented in a disaggregated way?** |  |  |  |  |

From: Andrade MV, Noronha K, Diniz BPC, Guedes G, Carvalho LR, Silva VA, Calazans JA, Santos AS, Silva DN, Castro MC. The economic burden of malaria: a systematic review. Malar J. 2022 Oct 5;21(1):283. doi: 10.1186/s12936-022-04303-6. PMID: 36199078; PMCID: PMC9533489.
