# Supplementary material for: Cost components of school-based oral health-promoting programs: A systematic review protocol
Source: PLoS One. 2023 Sep 28;18(9):e0287244. doi: 10.1371/journal.pone.0287244 (PMC10538792; doi:10.1371/journal.pone.0287244)
Supplement: S3 File — (DOCX) [file pone.0287244.s004.docx]

**S4- Data Collection Form**

| **Reviewer** | **Date** |
| --- | --- |
| **Author** | **Year** |
| **Journal** | **Record Number** |
| **Method of economic evaluation (CA/CM/CBA/CUA/CEA)** | |
| **Interventions** | |
| **Comparator** | |
| **Setting** | |
| **Geographical** | |
| **Participants/population (intervention/comparator, mean age, initial and final sample, condition, etc)** | |
| **Authors conclusions (factors that promote or impede the cost and effects of the intervention)** | |
| **Reviewers comments** | |
| **Funding** | |
| **Clinical Data** | |
| **Study design** | |
| **Source of data** | |
| **Analysis used** | |
| **Clinical outcomes results** | |
| **Economic Data** | |
| **Time horizon** | |
| **Analytical perspective** | |
| **Modeling (if used)** | |
| **Data source** | |
| **Collecting method** | |
| **Measure of benefit used in economic evaluation** | |
| **Direct costs (cost components)** | |
| **Indirect costs (cost components)** | |
| **Non health care costs** | |
| **Currency** | |
| **Year of reference** | |
| **Adjustments applied** | |
| **Discounts applied** | |
| **Statistical analysis** | |
| **Uncertainty treatment (sensitive analysis)** | |
| **Estimated benefits used in economic evaluation** | |
| **Cost results** | |
| **Synthesis of costs (resources used) and results** | |

**Outcome category ( )**

|  |  | **CLINICAL EFFECTIVENESS** | | |
| --- | --- | --- | --- | --- |
|  |  | **Better** | **Equal** | **Poorer** |
| **COST** | **Higher** | **A** | **B** | **C** |
|  | **Equal** | **D** | **E** | **F** |
|  | **Lower** | **G** | **H** | **I** |

From: Gomersall JS, Jadotte YT, Xue Y, Lockwood S, Riddle D, Preda A. Conducting systematic reviews of economic evaluations. Int J Evid Based Healthc. 2015;13(3):170-8. JBI. The Systematic Review of Economic Evaluation Evidence. In: JBI Manual for Evidence Synthesis [internet]. Adelaide: JBI. 2014 [cited 2023 ap. 8]. Available from: [https://synthesismanual.jbi.global](https://synthesismanual.jbi.global/).
